# Solving Challenges in Microalgae-Based Living Materials

**DOI:** 10.1021/acssynbio.4c00683

**Published:** 2025-02-21

**Authors:** Friedrich Hans Kleiner, Jeong-Joo Oh, Marie-Eve Aubin-Tam

**Affiliations:** Department of Bionanoscience, Kavli Institute of Nanoscience, Delft University of Technology, Van der Maasweg 9, Delft 2629 HZ, The Netherlands

**Keywords:** Microalgae, photosynthesis, living hydrogel, engineered living materials, stress responses, genetic modification

## Abstract

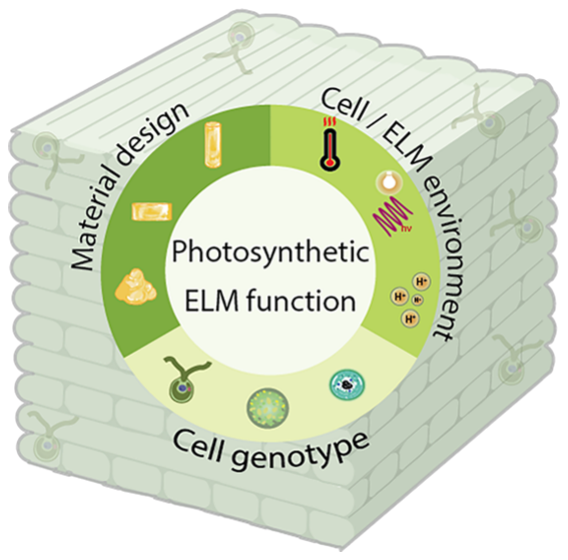

Engineered living materials (ELMs) integrate aspects of material science and biology into a unique platform, leading to materials and devices with features of life. Among those, ELMs containing microalgae have received increased attention due to the many benefits photosynthetic organisms provide. Due to their relatively recent occurrence, photosynthetic ELMs still face many challenges related to reliability, lifetime, scalability, and more, often based on the complicated crosstalk of cellular, material-based, and environmental variables in time. This Viewpoint aims to summarize potential avenues for improving ELMs, beginning with an emphasis on understanding the cell’s perspective and the potential stresses imposed on them due to recurring flaws in many current ELMs. Potential solutions and their ease of implementation will be discussed, ranging from choice of organism, adjustments to the ELM design, to various genetic modification tools, so as to achieve ELMs with longer lifetime and improved functionality.

## 1 Introduction to Microalgae-Based Engineered Living Materials

In so-called *engineered living materials* (ELMs), living cells constitute an intrinsic component of the material and impart a wide variety of life-like functions difficult to achieve in more traditional materials (e.g., sense-and-respond; self-repair; self-cleaning; or photo-, chemo-, thermo-, and mechanosensing functionalities). The term “engineered” herein applies to both the material and the organism. The cells within ELMs are typically immobilized in a matrix of biological or synthetic polymers. Functional living materials have been proposed for usage in a wide range of potential applications, including smart textiles and wearable devices,^1,2^ soft robots and actuators,^3^ and methods to control materials’ growth and mechanical properties.^4−7^

Microalgae are a highly diverse polyphyletic group of photosynthetic pro- and eukaryotes. They are important primary producers for many ecosystems,^8−12^ and jointly with plants they are responsible for the oxidizing atmosphere many current life forms depend on. With the urgent need to face the manifold environmental problems caused by anthropogenic activities, microalgae are investigated as a sustainable alternative to produce fuels, food products, or high-value metabolites.^13−15^

**Figure 1:**
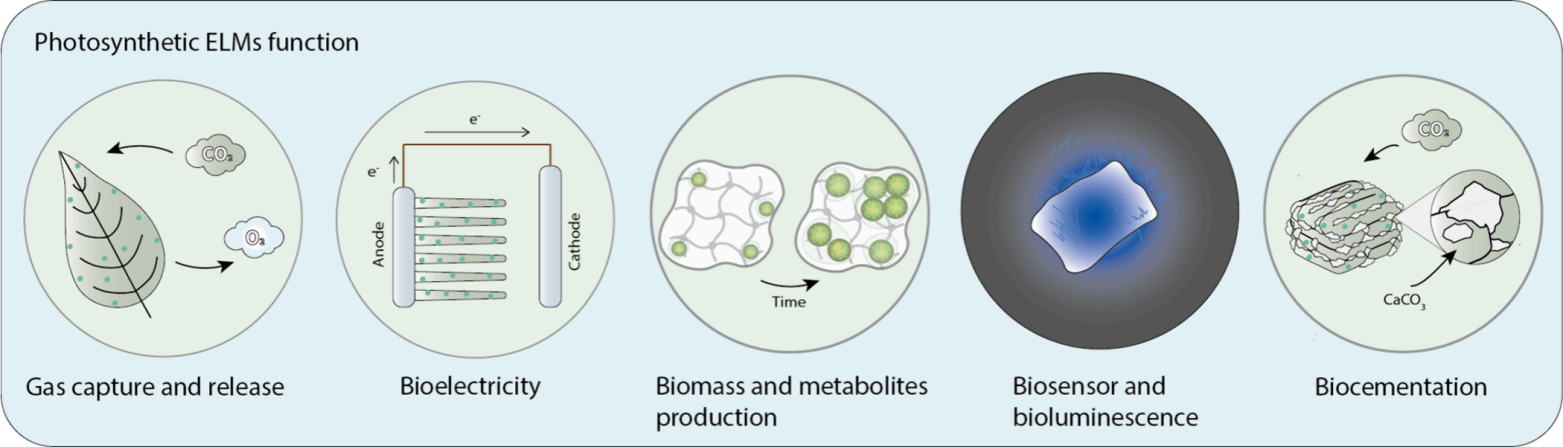
Representative functions of photosynthetic ELMs. When designing photosynthetic ELMs, the ability to fix CO_2_ often represents the primary motivation. This can also be combined with other functions such as electricity generation, growth platforms, biosensing, or the creation of novel regenerative materials.

Of particular interest are ELMs containing microalgae, which endow photosynthetic function to the material (Figure 1).^16−19^ Based on their ability to capture and produce gases (CO_2_, NO_X_, SO_X_ and O_2_, H_2_, respectively), photosynthetic ELMs are explored to treat wastewater,^20^ air, or soil^21^ and to deliver O_2_ to engineered tissues, organoids, or wounds.^22^ Inspired by liquid culture systems, they are also explored as a simple production platform for fatty acids,^23^ nutritional additives (e.g., vitamins, antioxidants), or carbonate minerals.^5,24^ Moreover, the electrons generated in the photosynthetic light reaction may be utilized in biophotovoltaic devices.^25^

### 1.1 Advantages of ELMs as Growth Platform

In most microalgal ELMs, cells are captured in flexible hydrogels, which present several advantages as a simple growth platform that can hold water and nutrients. To some degree, hydrogels mimic biofilms found in nature: Both form a physical barrier, which shields the cells from predatory organisms, slows down diffusion processes of toxins, and may absorb mechanical stresses through their viscoelastic properties. Immobilized microalgae are more tolerant to harmful conditions such as high light, grazers,^26^ high temperature,^19^ heavy metals,^27^ or pH,^28^ and often show higher cell numbers per volume compared to free growth conditions.^19,29−31^ However, while hydrogels include only a few main ingredients, natural biofilms additionally contain diverse EPS (extracellular polymeric substances), extracellular DNA, and enzymes (e.g., proteases) thought to intercept and defuse harmful agents such as reactive oxygen species,^32^ antibiotics, toxins, or enzymes (e.g., lysozyme).^33^ It remains to be explored in which way cells in ELMs “customize” their ELM environment in time, for instance through the secretion of proteins or EPS.

Growth in hydrogels also comes with practical benefits, such as reduced water use compared to liquid culture,^29,34^ improved handling (transport, harvest),^35^ and potentially more efficient light management.^18,36^ Some hydrogels also allow control over 3D shapes,^17,37,38^ further improving space and light management.

**Figure 2:**
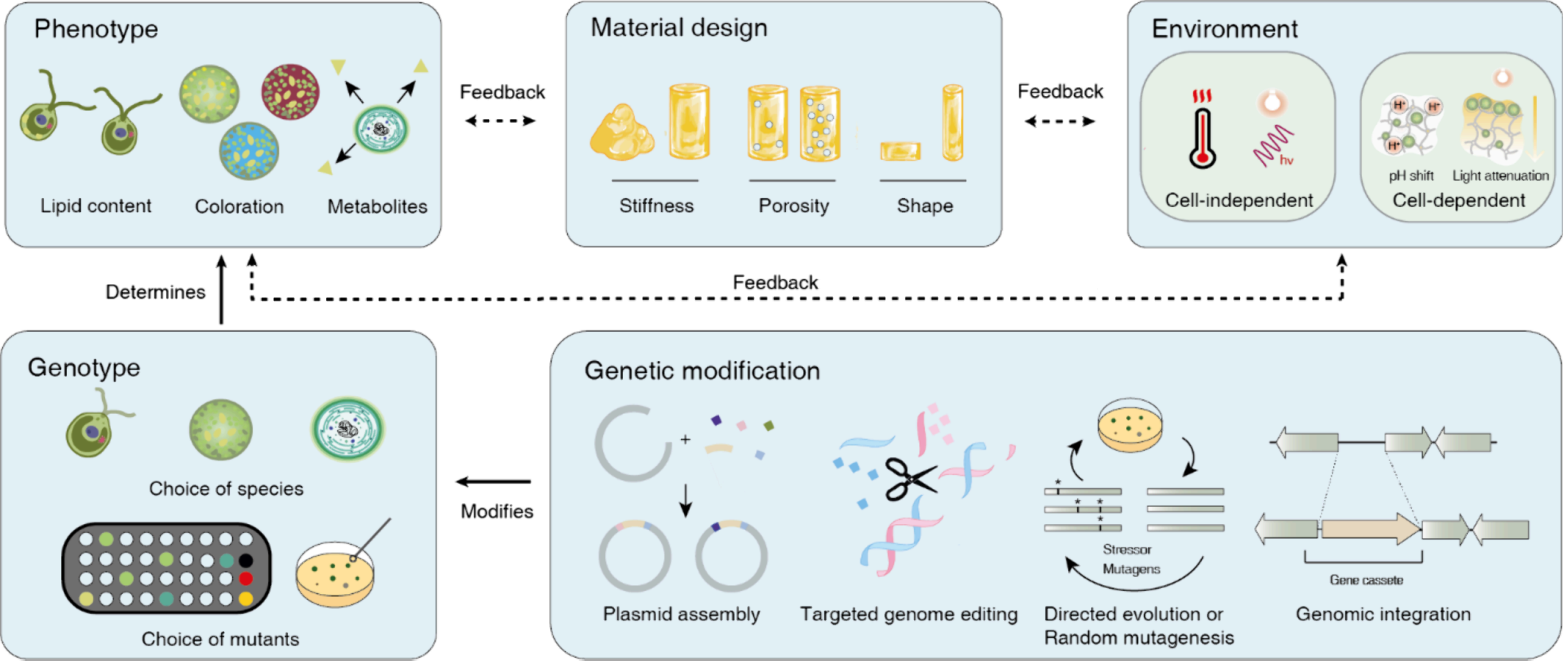
Challenges of ELMs: a complex communication of variables. Within an ELM, the phenotype of a cell (top left; lipid content, growth rate, etc.) is dependent on the environment into which an ELM is placed (top right; temperature, light, humidity, etc.). The perception and impact of environmental variables is moderated by design choices of the material (top middle; surface to volume ratio, water retention, etc.). The longer the duration, the more feedback occurs between cells, environment, and material (dashed arrows), potentially leading to new phenotypes. The genotype of the employed species with its inherent capacities and tolerance levels defines the type, onset, and scope of a phenotype (bottom left; lipid content, salinity tolerance, bioluminescence, etc.). The genotypes may also be expanded through genetic modification tools (bottom right; mutagenesis, genetic engineering, etc.). All these variables will influence the efficiency, reliability, lifetime, and thus the functionality of an ELM.

Despite their advantages, microalgal ELMs face many challenges. In this Viewpoint, we discuss variables that affect the functionality of ELMs (Figure 2), i.e., environmental variables like hydration or illumination; materials’ design variables like porosity or mechanical properties; and phenotypic variables (also linked to genotype) like lipid content, reactive oxygen species (ROS) production, or growth. Finally, we briefly discuss the potential avenues to address these challenges, including various genetic modification tools.

## 2 Challenges Faced by Microalgal ELMs

### 2.1 The Environment of an ELM Sets the Scene

The wide range of ELM applications is matched by a wide range of potential environments they are applied in, dictating ELM performance and lifetime. The scope of this Viewpoint does not allow an in-depth communication of the effects of each individual environmental factor, but a few examples will be mentioned. For instance, ELMs may be exposed to air^39^ or submerged in liquid. Air exposition challenges ELM integrity and viability through shrinking and desiccation,^40^ while submerged ELMs may swell or disintegrate at the periphery, releasing cells to the environment.^41^ The medium may be based on fresh- or saltwater, with ionic strengths that often dynamically change when hydration levels change. Since algae media are mainly composed of salts, ionic strength increases when water evaporates from the ELM. This imposes hyperosmotic stress on cells which can be attenuated but within limits through production of osmolytes. Moreover, shifted electrochemical gradients and increased abundance of competing substrates can affect selectivity of ion channels and nutrient-uptake transporters, respectively. From liquid-based algae farms we know that open growth conditions are cheap but are much more variable in light, temperature, and humidity; impose weathering; and are also more prone to contamination compared to closed growth conditions.^34^ Biocontaminants in ELMs may introduce symbiotic, commensalistic, parasitic, or competitive relationships.^42,43^ A symbiotic relationship is exemplified by a desired coculture of a green alga with a Gram-negative bacterium in a gelatin-based gel which led to 3-fold increase in algae growth-rate, 20% increase in photosynthetic activity, and lowered chance of further biocontamination after exposure to seawater.^43^ Parasitic or competitive relationships are regularly encountered but irregularly reported in the literature. It may hereby suffice to say that the environment in which an ELM is placed should be considered at the start of the ELM design phase.

### 2.2 Cell Phenotypes and Their Subject to Change

It is usually the phenotype of a cell which makes it attractive for a particular application, e.g. high lipid content,^44^ mechanosensitive bioluminescence,^45^ high electron export rates,^46^ fast growth,^47^*etc* (Figure 2). Applications generally favor reliability and consistency, which is not straightforward to achieve with living organisms that, based on their environment, may prioritize one biological activity over other ones. In excellent conditions, the major energy sink for an alga is usually growth. Suboptimal conditions will divert resources to lipid and antioxidant accumulation,^48,49^ protein repair processes,^50^ or maintenance of homeostasis^51^ or will lead to dormancy.^52^ It depends on the role of the ELM if any of these responses are desired. While high growth is desirable in applications focused on biomass accumulation, it is not required in applications that rely on basic cellular activity, such as O_2_ supply to wounds.^53^ Excess electrons in the photosynthetic light reaction may form ROS, harmful to any cells in proximity (e.g., other algae), but also form the basis for many biophotovoltaic devices. Compromises are likely inevitable, as exemplified with a desired high growth rate and lipid content in biofuels which is physiologically contradictory for most algae species. Admittedly, most of our physiological understanding of microalgae is based on liquid culture experiments. A better understanding of cellular behavior and growth in ELMs would allow designing ELMs to purposefully evoke desired phenotypes in microalgae (Figure 2).

Another potential problem of ELMs is its static nature. Lack of mixing and low gas diffusion rate is a bottleneck in liquid-pond culture productivity,^34,54^ and it is shown that bulky ELM designs suffer from similar problems.^17^ CO_2_ limitation shifts the carboxylase activity of RuBisCo toward the undesired oxygenase activity called photorespiration. In *Synechocystis* 20% of electrons generated by the photosynthetic light reaction are “wasted” in photorespiration at ambient-air CO_2_ conditions, which increases to 60% in carbon-limited conditions.^55^ This means that in bulky ELMs, the little amount of light penetrating to deeper layers may be wasted due to photorespiration. This is of special importance when artificial light is employed for growth. Moreover, if neither CO_2_ nor O_2_ are sufficiently accessible, fermentation processes with potentially problematic end products such as organic acids or ethanol may be activated.^56^ Sufficient gas exchange is therefore vital in photosynthetic ELMs.

The static nature may affect not only gases and solutes but also the cells. Many microalgae are biologically prepared to simply evade suboptimal conditions such as high light or low nutrients through swimming, gliding, or buoyancy, but in many ELMs cells are immobilized. Prolonged cellular metabolic activity will affect the surrounding microenvironment of single cells,^57,58^ and this process is likely accelerated when a high number of cells are placed within a small space with no mixing. Although this generally does not seem to trouble growth, microalgae in confinement often portray stress symptoms such as lipid or pigment accumulation, which could affect performance of some ELMs.^23,59−61^

There are multiple options to monitor algae cell stress levels. A key method already employed in several ELM publications is pulse-amplitude modulation (PAM) fluorometry on extracted cell suspensions, which is able to give insight into photosynthetic efficiency, electron transport rates, degree of nonphotochemical quenching, and more.^16,43,62^ PAM devices designed for leaves/tissues allow measurements at the ELM surface but may require the ELM to be relatively populated/“green”. O_2_/CO_2_ probes also give important information on productivity or gas levels and are especially insightful in combination with PAM-fluorometry measurements.^63^ Other indicators of stress are high ROS, low chlorophyll content, or elevated lipid/starch content, measured either directly, quantified indirectly through examination of related key enzymes, or visualized through fluorescence reporters.^64^ When faced with low growth rates, expression profiles of biomarkers related to uptake of a particular nutrient could reveal the growth-limiting factor.^64^ When faced with high mortality rates, measurement of caspase proteases could clarify whether the cause of death is stress-induced apoptosis as opposed to lysis caused by mechanical or osmotic stresses.^65^ However, many of those tools require access to cells, resulting in the loss of the ELM. Finding ways to apply some of these techniques without sacrificing the ELMs would help to determine occurrence of stresses in time.

### 2.3 The Issue of Duration

The longer the duration of an ELM application, the more the cell’s own metabolic activity impacts their environment (Figure 2). The most obvious example is light access, which negatively correlates with cell number due to self-shading effects.^17,18^ In addition, depending on the environment, the cell’s metabolic activity may eventually induce pH shifts,^66^ disadvantageous O_2_/CO_2_ levels,^17^ nutrient limitation, or accumulation of toxic waste products.^58^

Desiccation in air-exposed ELMs is of particular importance for both material and organism. Desiccation-induced shrinking can be associated with irreversible shifts in mechanical properties of the gel. Several pioneering hydrogel and bioconcrete ELMs require substantial moisturizing to ensure cell viability for more than a few days,^5,45,67^ which presents a serious drawback for most ELM applications. Organisms also contribute to dehydration through incorporation of H_2_O into new cell generations.

The pH of an ELM is a prime example of a cell-dependent variable (Figure 2). Continuous metabolic activity of microalgae in long-term ELMs will eventually induce pH shifts once the buffering capacity of the medium is exhausted, with implications for cell health and dissolved carbon chemistry. Shifts away from the cell’s respective pH optima are usually linked with increased lipid accumulation at the cost of biomass increase.^49,68,69^ For this reason, many photosynthetic bioreactors maintain stable pH through timed injection of CO_2_, removal of biomass, or other means.^66,70^ Compared to most liquid culture systems, subsequent adjustment of pH in ELMs is more difficult once they are fabricated.^66,71^

### 2.4 Limited Financial and Environmental Sustainability Data

Due to the relatively short time photosynthetic ELMs are subject of research, there is little data on scalability, cost, and quantitative environmental sustainability. We know from applied liquid cultures that techno-economic analysis is difficult due to the variety of production, maintenance, and harvest methods, leading to a wide range of production costs for microalgae (e.g., 2.9–290 €·kg^–1^).^72,73^ It is likely that ELMs have high production costs due to additional raw materials, manufacturing processes, and devices involved (e.g., gel ingredients, cross-linking agents, 3D printers). These would come on top of the more conventional costs, such as algae-growth and harvest prior to their integration into ELMs.

The environmental sustainability of photosynthetic ELMs is often praised; however, additional manufacturing and cell-extraction steps raise questions how sustainable some ELMs actually are. For instance, conventional purification of alginate or gelatin is associated with energy-intensive heating, drying, grinding, and sifting steps as well as substantial volumes of acidic and alkaline waste water.^74,75^

Although not every ELM application has economic interests in mind (e.g., medical applications) and many ELMs indeed have very unique properties difficult to realize otherwise (e.g., biosensors), aspects of economic and environmental sustainability could be presented more openly to allow techno-economic analysis and life-cycle assessment of their potential.

## 3 Possible Avenues to Solve Problems with Microalgal ELMs

### 3.1 Exploiting Natural Phenotypes

If stressful ELM conditions are inevitable, the choice of the organism becomes even more important. Assuming that a stressful ELM environment is temporary, employment of algae species which can switch to resilient but inactive morphotypes may be a solution.^76^ Examples include aplanospores (*Haematococcus*),^77^ akinetes (*Nostoc*, *Anabaena*),^52^ or cysts (*Euglena*).^78^

Another approach would be to anticipate the stressful environment and search for algae accustomed to similar natural habitats. For instance, estuarine waters are known for their wide range of osmotic conditions, and as such they host microalgae able to tolerate both salt- and freshwater to some degree.^79^ Examples for particularly exotic habitats include halophile,^80^ thermophile,^12^ or acidophile^81^ algae species. Many stressful habitats also induce coinciding tolerance strategies; for example, acidophiles often portray heavy-metal tolerance,^82^ or drought-tolerant desert algae portray exceptional tolerance for large diurnal shifts in temperature and illumination.^83^ Another potentially welcome side effect of extremophile algae is that the exotic conditions they thrive in are less prone to biocontamination.^84^

### 3.2 Exploiting the Flexibility of Material Engineering

The design and material composition of an ELM greatly influences how environmental stimuli are communicated to the cells (Figure 2). One potential advantage of ELMs is their high level of customizability. For example, we know a high surface area helps gas exchange and light penetration but is prone to water loss.^17^ Water-retaining coatings such as Latex or PDMS were shown to increase the lifetime of photosynthetic ELMs by weeks.^39,45^ To improve gas exchange and nutrient supply, ELMs were designed as small beads or to include hollow or sponge-like structures.^17,39,85^ As demonstrated in tissue engineering, vascular structures in ELMs could maintain water content and nutrient supply.^86^ Porous hydrogels filled with liquid would allow the cells to employ their motility and to direct the cell’s function into local areas.^87^ Details regarding material types, composition, printability, cross-linking types, surface adhesion methods, and degradability, of importance in the design of ELMs, can be found elsewhere.^88−90^

### 3.3 Can Genetic Modification Help to Improve Functionality?

If the environment or ELM design cannot be optimized further, and no suitable organism is easily found, a desirable phenotype may be obtained through the modification of the genotype (Figure 2). This may be achieved through adaptive laboratory evolution, mutagenesis, genetic engineering, or crossing.

#### 3.2.1 Adaptive Laboratory Evolution

In adaptive laboratory evolution (ALE), long-term exposure to specific growth conditions imposes selective pressure, driving genetic adaptation.^91^ In fact, ALE occurs naturally whenever cells are cultured in a lab environment. As such, ALE represents a low-cost, high-throughput technique which requires neither knowledge of the genome nor established genome editing tools. In microalgae, ALE led to strains with improved phenol biodegradation,^92^ thermotolerance,^93^ and lipid or pigment content.^94,95^ However, to manifest a new phenotype through mere stress exposure, multiple growth- and harvest cycles are required, which could be problematic to realize in ELMs.

#### 3.2.2 Mutagenesis

Mutagenesis is similar to ALE but accelerates the process by exposing organisms to physical or chemical mutagens.^96^ Like ALE, mutagenesis represents a GMO-greyzone in many countries since no foreign genes are introduced.^97^ In microalgae, mutagenesis has produced novel strains with enhanced lipid,^98,99^ pigment,^94,100,101^ or transgene expression,^102^ as well as improved tolerance to ELM-related stresses like salt-, temperature-,^103^ oxidative-,^104^ or alkali stress.^105^ Mutagenesis is therefore useful for identifying novel strains that tolerate ELM-associated stresses, including species lacking genetic tools.

Mutagenesis and ALE typically generate numerous mutants, thus requiring efficient high-throughput screening methods. While stress exposure represents a selection method on its own, increased productivity is more difficult to screen. A combined approach is shown by Laurent et al.^106^ who encapsulated single *Komagataeibacter sucrofermentans* cells after mutual UV- and stress exposure in gel beads including a cellulose-binding fluorescent dye. Through a microfluidic sorting system, single cells with superior cellulose production compared to those obtained with genetic engineering were identified.

#### 3.2.3 Genetic Engineering

In contrast to directed evolution and mutagenesis which usually aim to improve existing traits, completely novel properties may be introduced to microalgae via genetic engineering. In particular, genetic editing tools such as CRISPR and its emerging related technologies provide unprecedented opportunities, including CRISPR-associated transposons, integrases, epigenetic editing, and more.^107^ However, genetic engineering requires profound understanding of the genome, a transfection method in accordance with the cell’s features (e.g., cell walls), and an established selection method. This explains why relatively few algae species are currently being modified with sufficient reliability.^108^ Even successful transformation does not guarantee success, since obtained phenotypes are occasionally unstable due to eventual gene silencing, genome positioning effects, or overexertion of the metabolic burden.^108,109^

The variety of microalgae calls for individual transformation strategies. For example, there are notable differences between eukaryotes and prokaryotes to consider. Cyanobacteria have a simpler genome and cellular structure and can perform extrachromosomal replication/transcription of plasmids and their contents. However, their tendency for horizontal gene transfer might lead to biosafety concerns. Genetic engineering in eukaryotic microalgae is often more challenging. An extreme example are dinoflagellates with rampant retroposition in their huge, uniquely organized genomes, presenting problems even for genome sequencing.^110^ Expression of nonadjusted transgenes occasionally presents problems even in model organisms such as *C. reinhardtii*, here due to the intron-rich native gene structure.^111^ Some eukaryotic microalgae such as diatoms are diploid during their vegetative phase, which requires both alleles of a gene to be targeted when gene knockouts are intended. However, eukaryotic microalgae can offer flexibility through their compartmentalization, e.g., for storage of toxic recombinant gene-products in vacuoles. The available tools, purposes, and limitations for genetic modification in microalgae are reviewed elsewhere in more detail.^108^

Despite its limitations, genetic engineering is still highly relevant, e.g., for its ability to introduce completely novel functions, with biocontainment a useful example. One approach is to engineer microalgae to require a supplemented survival-factor, such as an essential nutrient or synthetic amino acid residue.^112^ An example would be *S. elongatus* growing on phosphite instead of the much more common phosphate through knock-in and knockout of respective uptake genes.^113^ Dependency on a survival-factor is considered genetically stable, since loss of this trait represents a disadvantage. Another approach is to engineer microalgae to sense a stimulus which is administered or present outside of their “permissive environment”, activating a suicidal kill-switch. This is exemplified by riboswitch-mediated autolysis of *S. elongatus* cells in response to theophylline.^16^ Kill-switches are considered genetically unstable, as it is in the interest of the organism to overcome its mortal susceptibility through mutation.^114^ As done in bacterial studies,^112,114^ a modular combination of biocontainment methods should therefore be considered for engineered microalgae in ELMs.

In algae, genetic engineering within applied context is mostly done to increase biomass^115,116^ or to increase production of a single valuable molecule, such as a fatty acid or pigment.^117−121^ This was achieved through constitutive expression of key enzymes,^119,122^ the interactors of key enzymes,^115^ knockout of competing pathways,^121^ or knockout of degrading enzymes specific for the desired product.^117^

Making algae more resilient to a changing environment encountered in long-term ELMs may be a more complex task compared to the increased production of a single metabolite. Inducible promoters would allow flexible expression of useful proteins in response to an environmental stimulus (temperature,^123^ light,^124^ salt,^125^ nitrogen compounds,^126^ phosphate,^118^ etc.) and could be the key to make algae either more resilient during stress or more productive during nonstress. Development of artificial promoters and riboswitches will expand the toolkit for inducible cell behavior considerably.^127^ However, genetic engineering may always come with unexpected side effects. To provide a speculative example, the isoelectric point of cell surface proteins in acidophile bacteria is relatively positive, thought to act as a natural charge-barrier for protons at the cell periphery.^128,129^ Using the right set of inducible promoters, algae could thus be engineered to temporally express such proteins at low pH. However, this may come with potentially relevant changes of resting membrane potential, membrane protein functionality, or cell–surface interaction.

#### 3.2.4 Crossing

Compared to mutagenesis and genetic engineering approaches, crossing is rarely mentioned as a solution for ELM-related challenges. Crossing is a powerful yet very simple tool, evident in the variety of agriculture crops generated by early humans without any understanding of genetics. Many microalgae are capable of sexual reproduction, with N-limitation induced syngamy in *C. reinhardtii* a prominent example. Oshima et al. crossed *C. reinhardtii* wild type cells with a cell-wall-less strain containing an ion-channel knockout, resulting in a daughter-strain with an intact cell wall but dysfunctional ion channel.^130^ The ability to simply combine beneficial traits of different strains is a tool worth considering for future photosynthetic ELM studies.

## 4 Outlook

While photosynthetic ELMs have promising and unique properties, they also present challenges related to long-term performance, reproducibility, scalability, etc. The efficiency of photosynthetic ELMs can be improved in various ways, such as adjustments in material design, environmental conditions, the choice of the organism, or its improvement through genetic modification tools. However, to apply these tools in a synergistic way, a more comprehensive understanding of the cell’s perspective in immobilized conditions is required. Then, scalable production of sustainable, efficient, and reliable ELMs containing genetically optimized microalgae will be possible.
